# Does Antibiotic Irrigation Really Reduce the Risk of Capsular Contracture of the Breast?

**DOI:** 10.1007/s00266-021-02456-4

**Published:** 2021-07-12

**Authors:** Eric Swanson

**Affiliations:** Swanson Center, 11413 Ash St, Leawood, KS 66211 USA

*Level of Evidence V* This journal requires that authors assign a level of evidence to each article. For a full description of these Evidence-Based Medicine ratings, please refer to the Table of Contents or the online Instructions to Authors www.springer.com/00266.

Awad et al. [1] report a systematic review comparing capsular contracture rates with the type of pocket irrigation. The authors conclude that antibiotic irrigation leads to significantly fewer capsular contractures than saline irrigation. The authors acknowledge that two studies by Pfeiffer et al. [2] and Drinane et al. [3] failed to show a significant advantage for antibiotic pocket irrigation compared with saline. However, with the addition of a cohort of patients, or rather breasts, published in an article by Burkhardt et al., [4] the authors (incorrectly) conclude that the overall rate of capsular contracture was significantly lower in the antibiotic group.

It is difficult to compare this study by Burkhardt et al. [4] with the studies by Pfeiffer et al. [2] and Drinane et al. [3] because capsular contractures are reported by breast rather than by patient and each patient received two different treatments. It is therefore necessary to convert the complication rates reported in these more recent studies to per-breast rather than per-patient. Pfeiffer et al. [3] reported 12 capsular contractures among 203 women (406 breasts) who received pocket antibiotic irrigation versus 17 capsular contractures among 211 women (422 breasts) who received saline pocket irrigation. Drinane et al. [4] reported 2 capsular contractures among 27 women (54 breasts) receiving antibiotic irrigation and 2 capsular contractures among 28 women (56 breasts) treated with saline irrigation. The difference in risk for both studies, whether considered individually or combined, is nonsignificant [1].

How is this comparison affected when the data from Burkhardt et al. [4] are added? Burkhardt et al. [4] reported 28 capsular contractures among 144 breasts that received some combination of antibiotics and steroids (19%) versus 15 capsular contractures among 37 control breasts (41%). Pooling the data from the 3 studies, [2-4] a total of 42 capsular contractures were reported for breasts treated with antibiotic solutions among 604 breasts (7.0%). By comparison, 34 capsular contractures were reported among 515 breasts that received either saline irrigation or no irrigation (6.6%). Control breasts actually experienced a slightly lower percentage of capsular contractures overall. A Chi-square test [5] produces a nonsignificant *p* value of 0.82 for this comparison (Table 1).

**Table 1:** Comparison of capsular contracture rates in 3 published studies of breast augmentation

Variable	Pfeiffer et al [2]	Drinane et al [3]*	Burkhardt et al. [4]	Combined
Antibiotic irrigation				
Capsular contractures	12	2	28	42
Patients (%)	203 (5.9)	27 (3.7)	Unknown	–
Breasts (%)	406 (3.0)	54 (3.7)	144 (19.4)	604 (7.0)
No antibiotic irrigation				
Capsular contractures	17	2	15	34
Patients (%)	211 (8.1)	28 (3.6)	Unknown	–
Breasts (%)	422 (4.0)	56 (3.6)	37 (40.5)	515 (6.6)
* p* (per breast)	NS	NS	< 0.01	NS

NS, not significant (*p* > 0.05)

*One patient in each group had a bilateral capsular contracture

Awad et al. [1] also compared antibiotic irrigation with no irrigation [1]. Only two studies included such a comparison [7, 8]. In one study, by Blount et al. [7], women receiving antibiotic irrigation experienced a 0.4% rate of capsular contracture versus 3.9% for women treated with no irrigation, a 10-fold difference. However, Blount et al. [7] found that there was no significant difference in capsular contracture rates on multivariate analysis (i.e., after correction for other variables impacting the capsular contracture rate). Giordano et al. [8] reported an advantage for antibiotic irrigation plus povidone-iodine solution, but the sample size (*n* = 330) was insufficient for a reliable conclusion [9].

The value of antimicrobial pocket irrigation has been challenged [10]. Bottles of Betadine (10% povidone-iodine) are labeled “Topical Bactericide.” The warnings, “Antiseptic Non-Sterile Solution” and “For External Use Only,” appear on the bottles [11]. Adams and Calobrace [12] recently suggested that the inside of the bottle is sterile, if not the outside. This statement is at odds with a communication released by the U.S. Food and Drug administration (FDA) [13].

In 2013, the FDA addressed safety issues regarding over-the-counter topical antiseptic products after receiving reports of infections that were confirmed to have been caused by contaminated topical antiseptics, including iodophors [13]. This issue was also reviewed in an article published in the *New England Journal of Medicine* in 2012 [14]. The FDA requests that manufacturers package antiseptics indicated for preoperative skin preparation in single-use containers. Health care professionals are instructed not to dilute antiseptic products after opening them. The FDA requests that manufacturers indicate on labels whether the antiseptic is manufactured as a sterile or nonsterile product. Topical antiseptics are not required to be manufactured as sterile and may become contaminated with bacteria during manufacturing. Labels that state that a product is sterile mean it was treated with a process during manufacturing to eliminate all potential microorganisms [13]. The FDA cautions, “If a product does not state ‘sterile’ on the label, health care professionals should be aware that they are using a nonsterile product” [15]. According to the FDA, all containers of Betadine 10% are nonsterile [16]. Only the 5% povidone-iodine ophthalmic solution, produced by another manufacturer and labeled “sterile,” is sterile [17].

Betadine is intended as a preoperative skin preparation, [11] not a solution to be poured into an open surgical wound. Decanting Betadine onto the surgical field from a bottle that has been opened multiple times is not recommended [18]. The fact that this topical antiseptic is intended for single use or comes in a sterile prep kit does not mean that the product is sterile.

Regardless of its antiseptic properties, any nonsterile solution introduced into a wound creates a risk of contamination by nonresident organisms [10, 13–15]. Guidelines published in *Annals of Surgery* warn that povidone-iodine solution is ineffective in decontaminating wounds, inhibits wound healing, and may increase the risk of wound infection [19]. These guidelines recommend against its use [19]. Fibrinogenic and proinflammatory antimicrobials that are used for pocket irrigation have been linked to an increased incidence of capsular contracture [20]. Some investigators dispute such deleterious effects and point to the absence of a definitive study in the context of a breast implant [18].

Jewell reports a personal experience of no breast implant infections using Betadine irrigation, and a 2% capsular contracture rate [18].

Jewell and Adams [18] believe that antibacterial irrigation is an essential part of a 14-point plan to reduce the risk of infection, capsular contracture, and even Breast Implant-Associated Anaplastic Large-Cell Lymphoma (BIA-ALCL). However, the only known factor associated with BIA-ALCL risk is textured implants, [21] which is not on the list. Jewell and Adams were co-authors of a large study that reported a 2.2% capsular contracture rate among authors using the 14 points [22]. No information was provided regarding how this rate was calculated, follow-up times, or numbers, and in fact, these 14 points were not consistently followed by the study authors [21]. Van Natta [23] recently commented: “Presumptively assuming that if the patients haven’t returned, then nothing must be wrong and therefore no cases of BIA-ALCL have occurred is not rigorous science and is certainly not valid data for comparison.” Notably, a 2.3% capsular contracture rate was also reported in another large study published by plastic surgeons who did not follow the 14 points and did not consistently use antimicrobial irrigation [24].

In contrast to the findings of this review by Awad et al., [1] 3 recently published systematic reviews, [9, 20, 25] including a meta-analysis [20] find no benefit for antibiotic irrigation or povidone-iodine irrigation. Swanson discontinued any form of antibiotic irrigation in favor of sterile saline irrigation without a change in capsular contracture rate (6%) [10, 26]. This experience compares favorably with the 8–19% rate of capsular contracture cited in core studies, [10] which are regarded as most robust. Using saline alone for pocket irrigation, two infections were encountered among 522 consecutive breast augmentation patients (0.4%) [26]. Other surgeons using saline for pocket irrigation also report low capsular contracture rates [27].

When analyzing study results, it is important to recognize sources of bias. Confirmation bias leads investigators to find in favor of an outcome that conforms to their hypothesis [28]. Successful outcomes in patients treated with saline irrigation alone [26, 27] may be overlooked. Publication bias makes it more likely that studies with positive findings (i.e., a benefit for antimicrobial irrigation) are published.

A 2018 systematic review co-authored by Thoma, [9] using the same Methodological Index for Non-Randomized Studies (MINORS) scale used by Awad et al., plus the Cochrane risk-of-bias tool revealed poor methodologic quality and selection bias among a heterogeneous patient population, predisposing to an erroneous association between antibiotic irrigation and capsular contracture.

In vitro effectiveness does not necessarily translate into clinical efficacy [10]. Breast surgery is different from many other types of surgery (orthopedics, for example) in that the wound environment is clean-contaminated rather than sterile [10]. Numerous commensal species are present, and it is impossible to eliminate them simply because the antibiotic solution cannot permeate all the breast tissue and the exposure to the dilute antibiotic solution is very brief. The use of antibiotics cannot be considered entirely innocuous. Eradicating commensal bacteria may be harmful; this practice may lead to an opportunistic infection [10].

One recent study using next generation sequencing, an evolution of polymerase chain reaction technology, reported microbial DNA in 42% of capsular contracture specimens and 120 unique bacterial species [29]. No control group was studied. A specific microbiome or organism has not been linked to capsular contracture. Bacterial profiles appear to be patient-specific rather than disease-specific [30]. Until it is clear that there is an advantage for antibiotic irrigation, any discussion of the superiority of one type of antibiotic solution over another is a moot point.

To make an informed decision regarding implant pocket antibiotic irrigation, plastic surgeons need to know which microbes, *if any*, are implicated in capsular contracture [10]. Sorting out causation from correlation is notoriously difficult [30]. Until we learn otherwise, an unproven practice that has possible risks is best avoided [10]. The evidence must prevail over conventional wisdom. That is the essence of evidence-based medicine.
